# Mollaret’s Meningitis: A Rare Entity

**DOI:** 10.7759/cureus.15264

**Published:** 2021-05-26

**Authors:** Abhinav Sehgal, Esana Pokhrel, Walter R Castro, Christopher J Haas

**Affiliations:** 1 Internal Medicine, Georgetown University School of Medicine, Washington, DC, USA; 2 Internal Medicine, MedStar Franklin Square Medical Center, Baltimore, USA

**Keywords:** mollaret's meningitis, aseptic meningitis, recurrent, benign aseptic meningitis, diagnosis, treatment, prognosis

## Abstract

We report on a patient with Mollaret’s meningitis to highlight the appropriate diagnostic criteria and benign prognosis without empiric antiviral therapy. An 83-year-old man with a history of aseptic meningitis of unknown etiology followed by full recovery presented with a two-day history of fevers, generalized weakness, and neurologic abnormalities. Cerebral spinal fluid (CSF) analysis demonstrated lymphocytic pleocytosis consistent with aseptic meningitis. Given his prior noninfectious aseptic meningitis and symptom-free interval, Mollaret’s meningitis was suspected and empiric treatment for herpes simplex viruses (HSV) encephalitis with acyclovir was deferred. All CSF studies, including polymerase chain reactions for HSV-1 and HSV-2, returned negative with clinical improvement by the fourth day of admission. For patients suspected to have Mollaret’s meningitis, lumbar puncture should be conducted promptly to facilitate diagnosis. Although several reports describe patients with CSF infection, the diagnosis of Mollaret’s meningitis should be reserved for noninfectious cases. In such cases, empiric antiviral therapy for HSV encephalitis may be deferred and complete recovery is expected.

## Introduction

Mollaret’s meningitis is an uncommon subtype of aseptic meningitis characterized by recurrent episodes of meningismus [1]. Classically, patients present with subacute to acute onset of fever with head and neck pain that may be accompanied by neurologic deficits. Cerebrospinal fluid (CSF) analyses during attacks often demonstrate lymphocyte-predominant pleocytosis, negative cultures, negative polymerase chain reactions (PCRs) and serologies for viral etiologies, and a normalization of the noted lymphocytosis in between attacks [2-4]. These symptoms spontaneously resolve without the need for antibiotic or antiviral therapy. Furthermore, empiric antiviral therapy for the herpes simplex virus (HSV) encephalitis prior to the return of CSF PCRs for HSV-1 and HSV-2 may be unnecessary in patients with aseptic meningitis who have history of recurrent noninfectious aseptic meningitis. Although flares of Mollaret’s meningitis can be severe and recurrent, the prognosis is favorable, with a lack of neurological sequalae following recovery.

## Case presentation

An 83-year-old man with a past medical history of sick sinus syndrome status post pacemaker placement, essential tremor, and an isolated episode of aseptic meningitis three years prior presented with a two-day history of fever, generalized weakness, and worsening essential tremor of the right hand. He reported usual health until two days prior to admission, at which time he developed sudden-onset severe generalized weakness that was followed by low grade fever over the next 24 hours. At baseline, per family report, the patient was an otherwise healthy, independent, and pleasant gentleman. Home medications prior to admission included: propranolol, primidone, rosuvastatin, digoxin, melatonin, acetaminophen, and alprazolam, without any recent medication changes or use of over the counter or herbal supplements. There was no noted recent travel, sick contacts, or additional exposures. On presentation he was febrile at 102°F and hypertensive at 161/84 mmHg with a regular heart rate (69 beats per minute) and preserved oxygen saturation on room air. Physical examination was notable for a pronounced oropharyngeal tic that abated with tongue protrusion and speech and fine bilateral distal upper extremity intention tremor. Muscular strength of the bilateral upper and lower extremities was nevertheless preserved (5 out of 5) and sensory function remained intact. Reflexes were brisk (2+) in all extremities and Babinski sign was negative bilaterally. In general, he remained awake, alert, and oriented to person, place and time with no focal neurologic deficits; however, he had fluctuating episodes of confusion and agitation during admission.

**Figure 1:**
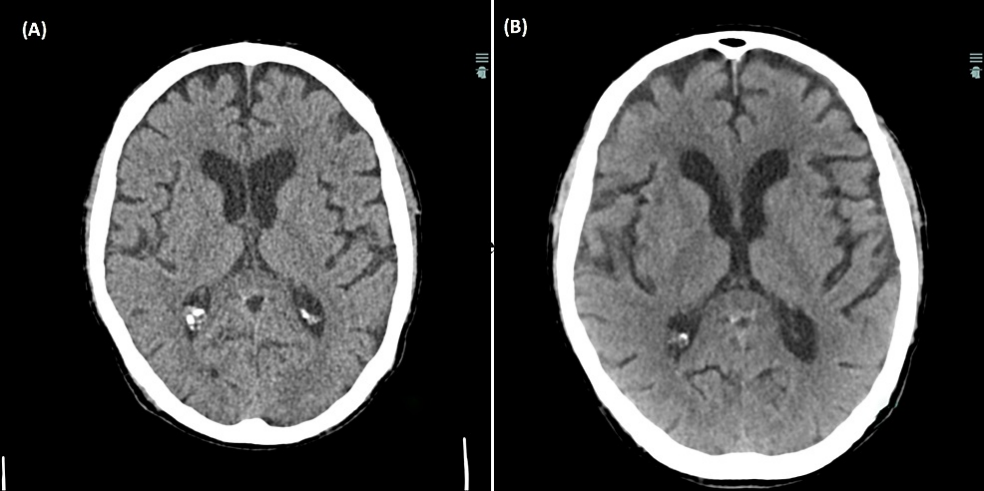
Computed tomography of the head without contrast from the 2017 admission (Panel A) and 2020 admission (Panel B).

Initial diagnostic evaluation included a complete blood count which demonstrated a preserved white blood cell count (6.6 k/uL) with a normal differential, hemoglobin (13.2 gm/dL), and platelet count (219 k/uL). Serum chemistries were within normal limits. Urinalysis and urine toxicology were unremarkable. Diagnostic imaging including a chest radiograph, computed tomography (CT) of the chest with contrast, and CT of the head without contrast (Figure 1) were unremarkable. COVID-19 and influenza virus testing were negative. Blood cultures were drawn and empiric therapy with vancomycin and piperacillin-tazobactam was initiated, with subsequent transfer to the medicine wards.

Of note, the patient had a similar presentation to our facility three years prior in 2017, presenting with fever, generalized weakness, and delirium. At that time, symptoms also commenced two days prior to admission with flailing myoclonic-like arm movements, culminating in a mechanical fall. At that time, he was empirically started on acyclovir and ceftriaxone for altered mental status and fever along with azithromycin (with concurrent ceftriaxone) for possible left lower lobe pneumonia. Lumbar puncture (LP) was performed with CSF analysis consistent with aseptic meningitis (Table 1), and acyclovir was stopped upon CSF studies, including HSV-1 and HSV-2 PCRs, returning negative (Table 2). He showed symptomatic improvement by the sixth hospital day and was discharged to subacute rehabilitation where he recovered to his functional and neurological baseline.

**Table 1:** CSF findings from the patient’s two admissions consistent with aseptic meningitis. CSF: cerebrospinal fluid; LP: lumbar puncture; WBC: white blood cell

Hospital Admission (Tube 4 of LP)	WBC (/mm^3^) Normal: 0-5 WBC	Lymphocytes (%)	Glucose (mg/dL) Normal: 40-70 mg/dL	Protein (mg/dL) Normal: <40 mg/dL
2017	100	48	54	77
2020	103	92	58	113

**Table 2:** CSF studies from the patient’s two admissions consistent with aseptic meningitis. IgG: immunoglobulin G; IgM: immunoglobulin M; DNA: deoxyribonucleic acid; PCR: polymerase chain reaction; RNA: ribonucleic acid; ELISA: enzyme-linked immunosorbent assay; HSV: herpes simplex virus; CSF: cerebrospinal fluid

CSF studies	2017	2020
Rickettsia IgG Rickettsia IgM		< 1:1 (Not detected)
Leptospira DNA - PCR		Not Detected
Enterovirus RNA - PCR	Not Detected	Not Detected
B. burgdorferi - ELISA	Not detected	Not detected
Cryptococcus Neoformans/gattii - PCR		Not Detected
Adenovirus DNA - PCR		Not Detected
Cytomegalovirus (CMV or HHV-5) - PCR		Not Detected
Escherichia coli K1 - PCR		Not Detected
Haemophilus Influenzae - PCR		Not Detected
Human Herpesvirus (HHV-6) - PCR		Not Detected
Human parechovirus (HPeV) - PCR		Not Detected
HSV1 DNA - PCR	Not detected	Not Detected
HSV2 DNA - PCR	Not detected	Not Detected
Listeria Monocytogenes - PCR		Not Detected
Streptococcus agalactiae - PCR		Not Detected
Streptococcus pneumoniae - PCR		Not Detected
Varicella Zoster Virus (VZV) - PCR	Not Detected	Not Detected
West Nile IgM		0.04 (negative)
West Nile IgG		0.05 (negative)
Bacterial culture	No growth	No growth

Throughout the current hospitalization, the patient remained hemodynamically stable. Laboratory diagnostics failed to demonstrate leukocytosis. Empiric antibiotics were discontinued on the second day of hospitalization and his blood cultures remained negative. In the setting of his presentation, physical examination, and unremarkable initial work-up, a presumptive working diagnosis of meningitis was made. A lumbar puncture was performed and CSF analysis once again demonstrated pleocytosis, elevated protein, and a normal glucose consistent with aseptic meningitis (Table 1). Neurology recommended acyclovir for empiric therapy of HSV encephalitis until viral PCR and CSF cultures returned, however acyclovir was deferred given high clinical suspicion of Mollaret’s meningitis. CSF viral PCRs, including HSV-1 and HSV-2, and serologies returned negative and cultures remained negative (Table 2). Head CT with contrast was performed (due to pacemaker-related MRI incompatibility) that demonstrated a stable, unchanged meningioma, previously seen on imaging (Figure 1). To further evaluate for a potential underlying malignancy contributing to a paraneoplastic disorder, CT imaging of his neck and abdomen, and bilateral renal ultrasound were performed, demonstrating the presence of only benign bilateral renal cysts, further lowering the suspicion for a paraneoplastic disorder. Additional investigation into autoimmune etiologies was deferred given his positive CSF findings consistent with aseptic meningitis and clinical improvement with conservative management by the fourth day of his hospitalization. Of note, the patient sustained a mechanical fall during hospitalization; however, thorough neurologic exam and CT imaging without contrast of the head following the fall event were benign. In the setting of his recurrent presentations, CSF studies demonstrating aseptic meningitis, and the low concern for an underlying infectious etiology, the diagnosis of Mollaret’s meningitis was made.

Similar to the 2017 admission, the patient’s presenting symptoms of fever and neurologic dysfunction resolved by the end of the hospital admission. Furthermore, the patient no longer demonstrated episodes of fluctuating cognition and returned to his baseline aware and pleasant demeanor. Given his significant clinical improvement by day four of admission without any neurologic sequalae, the patient was discharged to subacute rehabilitation for lingering weakness. On follow-up two months later, his next of kin shared that the patient had passed away at home from unrelated causes following return from subacute rehabilitation.

## Discussion

Mollaret’s meningitis has been reported to present abruptly with signs of meningismus such as neck stiffness and pain, headache, nausea, vomiting, photophobia, myalgias, and fevers [1, 2]. Multiple additional neurological symptoms were also noted - visual disturbance, speech impairment, facial paresis, pathologic reflexes, hallucinations, and coma [2]. Presenting symptoms were noted to resolve within days to several weeks without neurologic sequalae, specifically in the absence of antibiotic/antiviral therapy. Nevertheless, there appears to be an underlying susceptibility to recurrent attacks with marked variability in symptom-free intervals, ranging from a few days to years [3, 4]. Similar to previously published reports, the patient described in this report had recurrent, sudden-onset meningismus with associated neurologic symptoms that demonstrated complete resolution within one week without residual neurological sequelae and a noted prolonged recurrence-free period, consistent with the intervals noted in the literature [4].

First reported by Pierre Mollaret in 1944, the diagnostic evaluation of Mollaret’s meningitis revealed the presence of large mononuclear cells, termed “Mollaret cells” within the first 24 hours that is followed by lymphocytic pleocytosis within the CSF [5, 6]. Since then, immunocytological analyses with electron microscopy and immunohistochemistry demonstrated that Mollaret cells are monocytes with varying, but distinct morphologies including bilobed nuclei with a “bean-shaped” appearance, nuclei with a “footprint” appearance, cytoplasmic pseudopods, and degenerated cells termed as “ghost cells” found in over 90% of cases [7]. Mollaret cells are also characterized by their fragility and tendency to lyse, accounting for their rapid disappearance from the CSF and the subsequent lymphocytic predominance; thus, it is recommended to perform lumbar puncture promptly in those suspected to be having an attack [7]. Unfortunately, in our patient, lumbar puncture was performed at least four days after the onset of symptoms on both presentations with resultant CSF analyses demonstrating the presence only of lymphocytes, beyond the temporal window for visualization of Mollaret cells. Similar monocytic cells have also been described in the CSF of other pathologies such as sarcoidosis, Bechet’s disease, varicella zoster virus, herpes simplex virus, and West Nile virus, reducing the specificity of these distinctive cells for Mollaret’s meningitis [2, 3, 7-9]. However, the presence of Mollaret cells may still hold value as a sensitive diagnostic marker to support the diagnosis of Mollaret’s meningitis, provided the clinical picture is consistent and the distinguishing symptoms, diagnostics, and/or serologies of the aforementioned alternate pathologies are not present.

Given the clinical variability of Mollaret’s meningitis, as well as its similarity in terms of symptomology and late cytopathologic findings (e.g., lymphocytic pleocytosis) to other forms of aseptic meningitis, criteria have been proposed to aid in the diagnosis (Table 3).

**Table 3:** Proposed criteria for the diagnosis of Mollaret’s meningitis. CSF: cerebrospinal fluid

Bruyn et al. Criteria [3, 10]	Recurrent episodes of fever and meningismus; Episodes demonstrate CSF pleocytoses; Episodes are followed by symptom-free periods that last weeks or months; There are no sequalae or lingering symptoms following resolution of the episodes; No causative microbe is identified.
Galdi et al. Modified Criteria [3, 4]	Fever may not be present; In addition to meningismus, transient neurologic abnormalities may occur in approximately half of cases; Symptom-free periods between attacks range from days to years; CSF analysis may yield increased gamma globulin fraction.

Nevertheless, there are conflicting interpretations in the literature of the criteria regarding the presence of a causative microbe. Multiple case reports have described Mollaret’s meningitis in patients demonstrating recurrent aseptic meningitis in the context of an underlying detectable infectious etiology in the CSF, most commonly herpes simplex virus 2 (HSV-2) and less frequently, herpes simplex virus-1 (HSV-1) [1, 11]. A recent report by Willmann et. al. regarding a case associated with HSV-2 hypothesizes that a deficiency of toll-like receptors 3, involved in the innate immune system response to viral infection, may predispose to this recurrent lymphocytic meningitis [12]; however, whether this proposed pathophysiology can be extrapolated to reports of Mollaret’s meningitis without association with HSV-2 infection is unclear. Conversely, some reports adopt the stricter definition of benign recurrent aseptic meningitis that is idiopathic in nature, suggesting that Mollaret’s meningitis should be a diagnosis of exclusion [2, 5, 13]. Given Bruyn’s criteria regarding the absence of causative microbe, the diagnosis of Mollaret’s meningitis should be reserved for cases of attacks without detectable infectious etiology only [10]. The negative PCRs, including HSV-1 and HSV-2 PCRs, serologies, and culture results from the CSF from our patient’s recurrent admissions would further support this stricter definition of Mollaret’s meningitis.

While the underlying pathophysiology of the disease remains unknown, it has been hypothesized that intracranial cystic anomalies release squamous material resulting in meningeal irritation. However, evidence of this phenomenon can only be found on neuroimaging (CT head with contrast or nuclear magnetic resonance imaging of brain) when asymptomatic as these cysts collapse after releasing squamous material that precipitates attacks. Although neither of these studies were conducted for our patient, this etiology should be further explored with neuroimaging during asymptomatic intervals of patients with a history of Mollaret’s meningitis [14]. In addition, a causal relationship between nonsteroidal anti-inflammatory drug (NSAID) use and Mollaret’s meningitis has been proposed, suggesting an NSAID-induced hypersensitivity reaction that affects the meninges [15]. However, this patient did not use NSAIDs prior to either episode, making a mechanism involving this agent unlikely. In addition, one recent report highlighted elevated levels of cytokines such as interleukin-6 (IL-6) and tumor necrosis factor-alpha (TNF-α) in the CSF of a patient who demonstrated evidence of prior varicella zoster virus (VZV) infection (positive immunoglobulin G [IgG] and negative immunoglobulin M [IgM] serology on CSF analysis) without active infection. The report posited a mechanism in which subclinical viral infection may induce excessive cytokine release in the CSF [16]. Although the applicability of this proposed pathophysiology to our case is unclear in the absence of evidence of prior CSF infection, the reported finding of elevated cytokines in the CSF may warrant further evaluation. Another published report describes a case of infectious mononucleosis from confirmed Epstein-Barr virus (EBV) infection that was complicated by aseptic meningitis with negative EBV titers in the CSF, a complication reported to occur in less than 1% of primary EBV infections [17-19]. However, the patient continued to have recurrent bouts of aseptic meningitis, during which all serum and CSF cultures and serologies, including EBV titers, returned negative for active infection, leading to the diagnosis of Mollaret’s meningitis. The authors suggest that although the primary EBV disease-induced aseptic meningitis and the subsequent Mollaret’s meningitis may have been coincidental, the temporality of both disease courses may be indicative of recurrent subclinical reactivation of latent infection in the central nervous system (CNS) causing meningitis [17]. Unfortunately, serum viral serologies including that of EBV were not performed for our patient to assess for a prior peripheral viral infection that may be latent in the CNS and undetectable on CSF during episodes, however, this testing should be considered in those with Mollaret’s meningitis.

There is a lack of consensus regarding empiric treatment of suspected Mollaret’s meningitis in those with pending viral PCR results. As the clinical presentation and CSF findings of aseptic meningitis can be similar to that of HSV encephalitis, a life-threatening condition, there is a recommendation to empirically start antiviral therapy in those with consistent presentation and CSF findings to reduce morbidity and mortality associated with potential HSV encephalitis [20]. However, in this case and in other reports of Mollaret’s meningitis without causative organism, treatment consisted of supportive care with complete resolutions of illness absent of neurologic sequelae [2, 3]. Thus, for patients with a history of noninfectious Mollaret’s meningitis who present with meningismus and are found to have aseptic meningitis on CSF analysis, empiric antiviral therapy can be held and the patient may be managed with conservative care alone.

## Conclusions

Although Mollaret’s meningitis can present with severe recurrent meningismus and associated neurologic symptoms, the prognosis is excellent and without neurologic sequelae without the need for antiviral therapy. Individuals with a history of Mollaret’s meningitis who present with consistent symptoms should undergo prompt lumbar puncture and CSF analysis to visualize the distinct Mollaret cells and subsequent lymphocytic pleocytosis to facilitate the diagnosis. Diagnosis of this entity should be reserved for cases without detectable active infection of the CSF, and investigation of the etiology should be performed with neuroimaging to assess for cysts between attacks, inquiry of inciting drugs prior to the attack, and serum viral serologies to assess for latent subclinical infection of the CSF. Patients having an episode of Mollaret’s meningitis with pending viral PCR results do not require empiric antiviral therapy and may be managed with supportive care alone.
